# High-Quality Draft Genome Sequence of Kibdelosporangium philippinense, Generated by Hybrid Assembly of Short and Long Sequencing Reads

**DOI:** 10.1128/mra.00020-22

**Published:** 2022-04-04

**Authors:** Eugenia A. Fedorov, Medina Omeragic, Kristina F. Shalygina, Ashlyn C. Farwell, Kyle S. MacLea

**Affiliations:** a Biology Program, Department of Life Sciences, University of New Hampshire, Manchester, New Hampshire, USA; b Biotechnology Program, Department of Life Sciences, University of New Hampshire, Manchester, New Hampshire, USA; c Department of Life Sciences, University of New Hampshire, Manchester, New Hampshire, USA; University of Southern California

## Abstract

The glycopeptide antibiotic-producing soil actinobacterium Kibdelosporangium philippinense A80407 (=ATCC 49844) was sequenced using Illumina and Nanopore sequencing methodologies, and a hybrid genome assembly was generated for this type strain, with a total predicted genome length of 12,054,556 bp, 10,953 protein-coding sequences, 79 RNAs, 298 pseudogenes, and a G+C content of 65.13%.

## ANNOUNCEMENT

Kibdelosporangium philippinense A80407^T^ (=ATCC 49844^T^ = NRRL 18198^T^) is an unsequenced glycopeptide antibiotic-producing actinobacterium (1). Of the sequenced members of its genus, only Kibdelosporangium phytohabitans has a high-quality genome sequence available. Since *Kibdelosporangium* spp. produce numerous interesting antimicrobials (2–8) and have large genomes, averaging about 12 Mbp, sequencing other members of the genus is a priority.

For this study, freeze-dried *K. philippinense* ATCC 49844^T^ (ATCC, Manassas, VA, USA) was rehydrated and subcultured on International Streptomyces Project 2 (ISP2) medium (BD, Franklin Lakes, NJ, USA), with a single colony grown in ISP2 broth (Teknova, Hollister, CA, USA) at 30°C/1 atm for 240 h. Genomic DNA (gDNA) was isolated using the FastDNA Spin kit for soil (matrix A; MP Biochemicals, Irvine, CA, USA) for Illumina sequencing and the Nanobind CBB Big DNA kit (Circulomics, Baltimore, MD, USA) for Nanopore sequencing. The KAPA HyperPlus kit (KR1145, v.3.16; Wilmington, MA, USA) was used to generate the Illumina sequencing library. The Hubbard Center for Genome Studies (HCGS; Durham, NH, USA) sequenced this library on a HiSeq 2500 instrument, producing 250-bp paired-end fragments. Trimmomatic v.0.38 (9) was used to trim the resulting reads (settings: paired-end mode with a window size of 4, quality requirement of 15, and minimum read length of 36). A long-read Nanopore library was generated using the ligation sequencing kit (LSK109; ONT, Oxford, UK), run on a MinION R9.4.1 flow cell/GridION instrument (HCGS), and base called using the Guppy v.5.0.13 base caller (10) in SUP mode. Adapters were trimmed using Porechop v.0.2.4 (read setting: 1000; the Nanopore reads as analyzed by QUAST [11], yielded an *N*_50_ value of 17,277 bp), and the reads were filtered using Filtlong v.0.2.1 (settings, top 80% quality and length 1,000 bp). SPAdes v.3.13.0 (12) was used to assemble 2,926,840 trimmed short reads and 239,141 long reads with default bacterial assembly parameters in hybrid mode. Small contigs (<200 bp) and contigs with low coverage (<9.5×) were removed.

The assembly contained 27 contigs—the largest being 4,055,460 bp—with an *N*_50_ value of 2,839,569 bp (11). More than 94% of the genome size (12,054,556 bp) is comprised by just 4 contigs, and only 7 contigs of the assembly are larger than 500 bp. However, 200- to 500-bp contigs matching (via BLAST [13]) closely related species (*Kibdelosporangium* sp. strain MJ126-NF4 [GenBank accession number GCF_000826545.1], Amycolatopsis aidingensis [GCF_018885265.1], *Amycolatopsis* sp. strain CA-230715 [GCF_018736145.1], Saccharopolyspora pogona [GCF_014697215.1], Saccharopolyspora erythraea [GCF_002564065.1], and Saccharopolyspora spinosa [GCF_014490055.1]) were retained. It is unclear if the 4 largest contigs represent chromosomes; however, one of the *Kibdelosporangium* sp. genomes sequenced (Kibdelosporangium phytohabitans) does have a hybrid Illumina-PacBio assembly (GCF_001302585.1) that resolves into a single 11.75-Mbp chromosome. Therefore, we expect that these contigs represent large chunks of a single chromosome. We also calculated a G+C content of 65.13%. Although there is no previously published value, our calculation is close to that of the type member of the genus, Kibdelosporangium aridum, as determined chemically (66% ([2]) or *in silico* (66.2% [14]).

The genome is 99.1% complete according to an analysis using Benchmarking Universal Single-Copy Orthologs (BUSCO) v.5.2.2 (default parameters) (15), with an average genome coverage of 60.72×. The Prokaryotic Genome Assembly Pipeline (PGAP) (16) annotated 10,953 protein-coding genes, 298 pseudogenes, 79 RNAs, including 3 complete copies of the rRNA genes (also seen in the single-chromosome *K. phytohabitans* assembly above), 3 noncoding RNAs (ncRNAs), and 67 tRNAs. *Kibdelosporangium* spp. produce antimicrobials, and their genomes contain numerous biosynthetic gene clusters (BGCs) related to these metabolic pathways (1–6, 8, 14, 17). Forty-two BGCs were found in *K. philippinense* using antiSMASH 6.0 (18), including 26 nonribosomal peptide synthetase and/or polyketide clusters.

### Data availability.

The Kibdelosporangium philippinense ATCC 49844^T^ whole-genome shotgun sequence (WGS) project has been deposited at DDBJ/ENA/GenBank under accession number JAJVCN000000000. The raw data, found under BioProject accession number PRJNA790681, were submitted to the NCBI Sequence Read Archive (SRA) under three experiment accession numbers: SRX13551729 (Illumina fastq files), SRX13555714 (Nanopore reads in fastq format), and SRX13556647 (Nanopore reads in fast5 format).
